# Influenza A virus PB1-F2 protein prolongs viral shedding in chickens lengthening the transmission window

**DOI:** 10.1099/jgv.0.000584

**Published:** 2016-10-13

**Authors:** Joe James, Wendy Howard, Munir Iqbal, Venugopal K. Nair, Wendy S. Barclay, Holly Shelton

**Affiliations:** ^1^​ Avian Viral Diseases Programme, The Pirbright Institute, Pirbright, Surrey, UK; ^2^​ Faculty of Medicine, Imperial College London, Norfolk Place, London, UK

**Keywords:** PB1-F2, avian influenza, transmission

## Abstract

Avian influenza is a significant economic burden on the poultry industry in geographical regions where it is enzootic. It also poses a public health concern when avian influenza subtypes infect humans, often with high mortality. Understanding viral genetic factors which positively contribute to influenza A virus (IAV) fitness – infectivity, spread and pathogenesis – is of great importance both for human and livestock health. PB1-F2 is a small accessory protein encoded by IAV and in mammalian hosts has been implicated in a wide range of functions that contribute to increased pathogenesis. In the avian host, the protein has been understudied despite high-level full-length conservation in avian IAV isolates, which is in contrast to the truncations of the PB1-F2 length frequently found in mammalian host isolates. Here we report that the presence of a full-length PB1-F2 protein, from a low pathogenicity H9N2 avian influenza virus, prolongs infectious virus shedding from directly inoculated chickens, thereby enhancing transmission of the virus by lengthening the transmission window to contact birds. As well as extending transmission, the presence of a full-length PB1-F2 suppresses pathogenicity evidenced by an increased minimum lethal dose in embryonated chicken eggs and increasing survival in directly infected birds when compared to a virus lacking an ORF for PB1-F2. We propose that there is a positive pressure to maintain a full-length functional PB1-F2 protein upon infection of avian hosts as it contributes to the effective transmission of IAV in the field.

## Introduction

The natural reservoir for the first 16 haemagglutinin (HA) and 9 neuraminidase (NA) subtypes of influenza A viruses (IAVs) is the wild aquatic bird population, where infection is generally asymptomatic and reassortment between strains occurs regularly (Reperant *et al.*, 2012). Spillover of these viruses into domesticated poultry populations has resulted in the evolution of highly pathogenic avian influenza (HPAI) subtypes such as the Asian-lineage H5N1 which has been circulating continuously in birds since 2003 and has caused upwards of 700 human infections (To *et al.*, 2012). Low pathogenicity avian influenza (LPAI) H9N2 viruses are frequently found circulating in the same geographical areas as HPAI H5N1. HPAI H5N1 as well as LPAI H9N2 can cause a significant economic and welfare burden in geographical regions such as the Middle East and Southeast Asia. In these areas both subtypes are enzootic in the poultry populations making control costly and challenging (Alexander, 2007). As well as threatening food security, an increasing range of avian influenza subtypes causing zoonotic infections in humans is of considerable concern for public health agencies (Yu *et al.*, 2014). Increasing the fundamental knowledge base about avian influenza virus interactions within avian hosts will enable the design and implementation of better control strategies.

IAVs have a segmented ssRNA genome that encodes for a core repertoire of 10 viral proteins which all subtypes express, allowing successful viral replication. In addition there are a plethora of accessory proteins encoded by IAVs that are non-essential for replication, but may contribute to the virulence, pathogenesis and transmissibility of strains in different host species. The first of these so-called accessory proteins to be identified was the PB1-F2 protein (Chen *et al.*, 2001), which can range in full-length size from 90 to 101 aa and is translated in a +1 reading frame from the same viral mRNA that encodes the viral RNA-dependant RNA polymerase subunit, PB1.

Functional characterization of PB1-F2 has been undertaken in mammalian cell lines and hosts. However, in viruses isolated from mammalian hosts, humans and swine, PB1-F2 is often truncated as a result of mutations that generate stop codons in the PB1-F2 ORF (Krumbholz *et al.*, 2011; Zell *et al.*, 2007). More than half of all human and swine isolate sequences deposited in the public sequence databases encode a PB1-F2 less than 90 aa in length. In contrast, more than 90 % of sequences from isolates sequenced from avian hosts retain the coding capacity for a full-length PB1-F2 protein (Kamal *et al.*, 2015; Zell *et al.*, 2007). This disparity of PB1-F2 full-length conservation between virus isolates from mammalian and avian hosts suggests a unique pressure to maintain a full-length PB1-F2 protein with evolutionary significance in avian hosts. The most fundamental evolutionary pressure for IAVs is to transmit. To date there has been no robust assessment of the involvement of PB1-F2 in transmission of influenza viruses in any host species.

In mammalian IAVs, PB1-F2 has been reported to have a wide range of roles influencing pathogenesis including modulating the innate immune response (Le Goffic *et al.*, 2010), affecting activity of the viral polymerase (Mazur *et al.*, 2008) and increasing susceptibility to secondary bacterial infections (McAuley *et al.*, 2007). Work to delineate the function of avian influenza virus PB1-F2 in avian hosts has only been limited to highly pathogenic H5N1 influenza strains and these studies have concentrated on reading out pathogenic outcomes (Hulse-Post *et al.*, 2007; Leymarie *et al.*, 2014; Schmolke *et al.*, 2011). No publications have examined the role of PB1-F2 in a LPAI background, which make up the vast majority of avian influenza infections globally.

Avian influenza viruses rely on close mixing of infected and susceptible species and/or the sharing of common contaminated areas, such as water sources, for effective transmission of the virus either through aerosol or fomite ingestion (Breban *et al.*, 2009; Brown *et al.*, 2007; Hinshaw *et al.*, 1979). In this study, we utilize a LPAI H9N2 to assess whether PB1-F2 contributes to transmission dynamics in chickens. We measure infectivity, pathogenic outcomes as well as transmissibility using two different transmission scenario settings. The first scenario models transmission in a crowded, confined environment, representing a large commercial poultry shed. The second scenario models transmission that may result as a consequence of sporadic contact, reflective of behaviours in the natural avian species reservoir and incursions into traditional backyard flocks found abundantly in the Middle East and Southeast Asia where H9N2 viruses are enzootic.

In line with previous results seen investigating functions of PB1-F2 with HPAI viruses (Leymarie *et al.*, 2014), we observed a significant increase in pathogenicity when PB1-F2 was removed from our LPAI H9N2 virus, in an embryonated chicken egg model and in chickens *in vivo*. We also determined that the presence of PB1-F2 in an H9N2 virus prolonged the infectious shedding period in infected chickens which resulted in an extended transmission window with enhanced transmission.

## Results

### A full-length PB1-F2 protein is conserved at a higher rate in avian viral isolates compared to mammalian isolates

Previous analyses indicated that the full-length PB1-F2 ORF was more often conserved in IAV strains isolated from avian hosts than those isolated from mammalian hosts. To verify and extend these data, we downloaded all full-length PB1 influenza segment nucleotide sequences from the NCBI database for avian, swine and human hosts, removing duplicate sequences. Subsequently, 24 803 PB1 nucleotide sequences were analysed in the +1 reading frame to determine the length of the PB1-F2 ORF. We defined full-length PB1-F2 as 87 aa or longer since this is the length of the well-studied PB1-F2 protein of PR8 virus, and has been shown to enable mitochondrial targeting in all instances where it has been assessed. Other published analyses have used a PB1-F2 length of 79 aa or more (Zell *et al.*, 2007), based on the minimal length for an intact mitochondrial targeting sequence of porcine isolates in HeLa cells (Zell *et al.*, 2007), but this is a notional cut-off bearing in mind mitochondrial targeting has been seen for a PB1-F2 of only 75 aa for PR8 in Vero cells (Yamada *et al.*, 2004) vs 87 aa in HeLa cells (Gibbs *et al.*, 2003) and even then not all PB1-F2 proteins of length greater than 89 aa localize to the mitochondria (Chen *et al.*, 2010). However, a reanalysis of our data for length at least 79 aa indicates little alteration of the frequencies from using 89 aa or more (Table S1, available in the online Supplementary Material). The percentage of full-length PB1-F2 in human, swine and avian hosts was calculated as 43, 48 and 93 %, respectively (Table 1).

**Table 1.** Percentage of influenza A sequences possessing full-length PB1-F2 (≥87 aa) isolated from avian, human and swine hosts

Host	% Sequences with PB1-F2 ≥87 aa (*n*)*
Avian†	93 (10 401)
Anseriformes	97 (6917)
Galliformes	87 (1804)
Charadriiformes	98 (1298)
Human	43 (11 729)
Swine	48 (2673)
Total	100 (24 803)

*(*n*), Number of sequences.

†All avian orders.

### Generation of a recombinant virus lacking PB1-F2

**Fig. 1.**
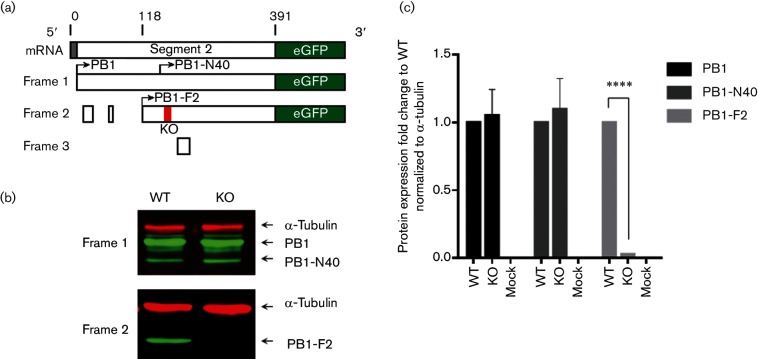
Segment 2 gene expression from UDL01 influenza virus. (a) Schematic of the ORF structure of 5′ end of UDL01 influenza virus segment 2 mRNA fused to eGFP in either frame 1 or frame 2. Nucleotide positions are indicated. Red bar indicates the position of a stop codon at aa position 12 of PB1-F2 in the KO UDL01. (b) Lysates from 293T cells harvested 48 h post transfection with indicated plasmids were detected via Western blot with anti-GFP with anti-α-tubulin as a loading control. (c) Levels of PB1-, PB1-N40- and PB1-F2-GFP from transfected lysates were quantified using densitometry. Means±sd were calculated from three independent transfections. Levels of significance were based upon *P*-values from a Student’s *t*-test; ****P<0.0001.

To understand the role of the PB1-F2 protein in the poultry host, we used a LPAI virus strain that is representative of a H9N2 major lineage (G1-like) that circulates in the enzootic Pakistan region of the Middle East (Iqbal *et al.*, 2013). A reverse genetics system for the A/chicken/Pakistan/UDL01/08 (UDL01) H9N2 strain used previously (Long *et al.*, 2016) was rescued as infectious virus using standard techniques (Hoffmann *et al.*, 2000). UDL01 contains a full-length 90 aa PB1-F2 protein encoded in the +1 reading frame of the PB1 segment, and this virus was designated as WT. Alongside the WT version we generated an isogenic virus that had a stop codon introduced at aa position 12 in the PB1-F2 ORF, producing a non-functional 11 aa PB1-F2 fragment, which we termed knockout (KO). The introduction of the stop codon did not alter the aa sequence of the PB1 reading frame. To assess if this method of PB1-F2 KO altered protein expression levels of either PB1 or PB1-N40 protein, we utilized a previously published method (Wise *et al.*, 2011) of tagging the different reading frames from which the mRNAs are transcribed (Fig. 1a). A plasmid was generated whereby the viral sequence, including the upstream non-coding region that the ribosome recognizes preceding the initiation codon for PB1, was cloned and two different constructs were generated in which GFP was placed in-frame at the C-terminus with either the PB1, PB1-N40 or in the +1 frame with the PB1-F2 message. Expression in human embryonic kidney (HEK) 293T cells and subsequent detection and densitometry quantification of the viral proteins expressed, indicated no alteration in the level of PB1 or PB1-N40 expression occurred when mutation of the +1 frame to KO PB1-F2 was introduced (Fig. 1b, c).

### PB1-F2 has no effect on viral replication of H9N2 virus in avian cells *in vitro*

**Fig. 2.**
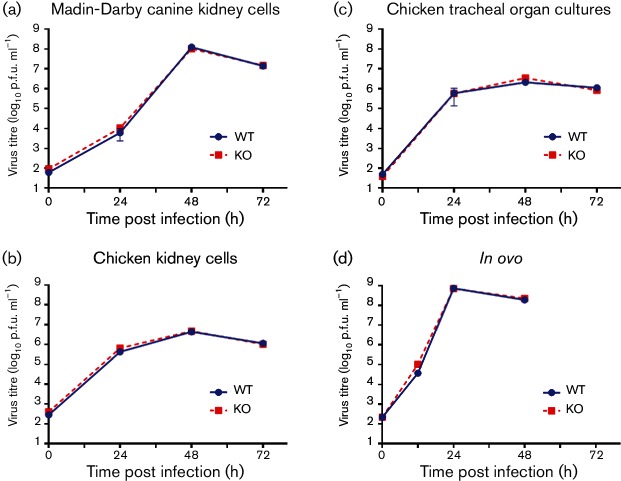
*In vitro* growth curves of UDL01 WT and KO viruses. Cellular substrates were inoculated at a low m.o.i. with either WT (blue circles) or KO (red squares) UDL01 influenza virus. The level of infectious virus was determined at times specified. Data represent the means of at least three independent experiments±sd. (a) MDCK cells and (b) primary cKCs were inoculated at an m.o.i. of 0.0001. (c) Chicken tracheal organ cultures were inoculated with 100 p.f.u. (d) The 10-day old Rhode Island Red chicken eggs were inoculated into the allantoic cavity with 100 p.f.u.

We assessed the ability of the isogenic H9N2 virus pair to undergo multiple cycles of replication from a low m.o.i. (0.0001 p.f.u.) in a range of avian cell substrates *in vitro.* No replication differences were found between the WT and KO H9N2 viruses in Madin–Darby canine kidney (MDCK) cells (Fig. 2a). Similarly no replicative differences between the two viruses were observed in primary chicken kidney cells (cKCs), *ex vivo* chicken tracheal organ cultures (cTOCs) and 10-day-old embryonated chicken eggs (Fig. 2b–d).

### Presence of a full-length PB1-F2 protein reduces pathogenic outcome of infection *in ovo*

During the growth curve analyses of the UDL01-WT and KO viruses, we observed that eggs infected with the KO virus were reaching terminal end points (no movement of the embryo or haemolysis of blood vessels) between 24 and 32 hours post infection (hpi)compared to 32–48 hpi for those infected with the WT virus. Therefore, we used a 10-day-old embryonated chicken egg model to assess the pathogenicity of the two viruses by performing a minimum lethal dose (MLD) assay. A 10-fold dilution series of each virus, WT and KO, was used to inoculate groups of embryonated eggs which were monitored twice daily for end point. The percentage death in each group of H9N2-infected eggs reached after 72 h was calculated for each dilution. The MLD was defined as the lowest viral dose resulting in death of all infected embryonated eggs by 72 h (Table S2). The presence of a full-length PB1-F2 in the WT virus resulted in a higher MLD (1 p.f.u.) compared to the KO virus (0.01 p.f.u.). The minimum infectious dose (MID) defined as the lowest viral dose required to produce infection in all embryonated chicken eggs was the same for both the WT and KO virus (0.1 p.f.u.). Having seen no difference in the magnitude or rate of infectious virus particle production in embryonated eggs, we wanted to determine if differing levels of defective viral particles were produced by the KO virus in comparison to the WT virus that could explain the increased pathogenicity. Quantitative reverse transcription PCR (qRT-PCR)to quantify influenza virus M gene viral RNA (vRNA) levels was performed on the allantoic fluid recovered after inoculation of embryonated eggs. We assessed three independent egg stocks and the genome copy number: particle forming unit ratio remained the same between the WT (14.5±2.13) and KO (12.39±4.37) viruses, indicating no difference in defective particle levels (Table S2). Another virus tested in parallel with an internal gene constellation of an HPAI H5N1 virus (A/chicken/England/50–92/91) had a significantly different genome : p.f.u. ratio (43±6.9) to the two H9N2 viruses.

### Presence of a full-length PB1-F2 protein reduces pathogenic outcome of infection for LPAI virus *in vivo*

**Fig. 3.**
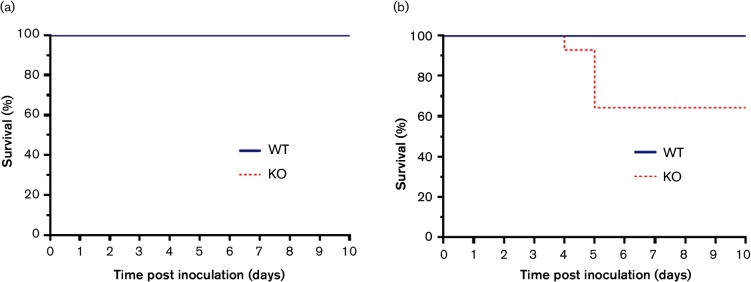
Survival curves of chickens inoculated with WT or KO UDL01 influenza virus. Survival curves of chickens inoculated into the nares with 100 µl of PBS containing (a) 5×10^3^ p.f.u. (low dose) or (b) 1×10^5^ p.f.u. (high dose) of either WT (blue line) or KO (red dashed line) UDL01 influenza virus.

Leymarie *et al.* (2014) previously reported that the removal of the PB1-F2 protein from an HPAI H5N1 virus resulted in an increase in chicken mortality at low dose. To determine if the absence of PB1-F2 also increased the pathogenicity of LPAI viruses in chickens, we inoculated groups of chickens via the nares with either a low dose (5×10^3^ p.f.u.) or high dose (1×10^5^ p.f.u.) of the UDL01-WT or KO virus. The chickens were monitored daily for clinical signs and scored accordingly. Infection of birds with WT virus produced transient clinical signs including red and watering eyes, swollen combs and swollen foreheads. No difference in clinical signs was observed in birds infected with the KO virus at the low dose. However, upon infection with the higher viral dose, 35 % (5/14) of the birds infected with the KO virus died between days 4 and 5 post inoculation. There was no significant difference in clinical score prior to death between the WT and KO groups. The remaining birds infected with the KO virus showed no ill effects out to day 14 post inoculation when the experiment was ended. No deaths were recorded for the group infected with WT virus (Fig. 3).

### A full-length PB1-F2 protein prolongs infectious virus shedding from chickens

**Fig. 4.**
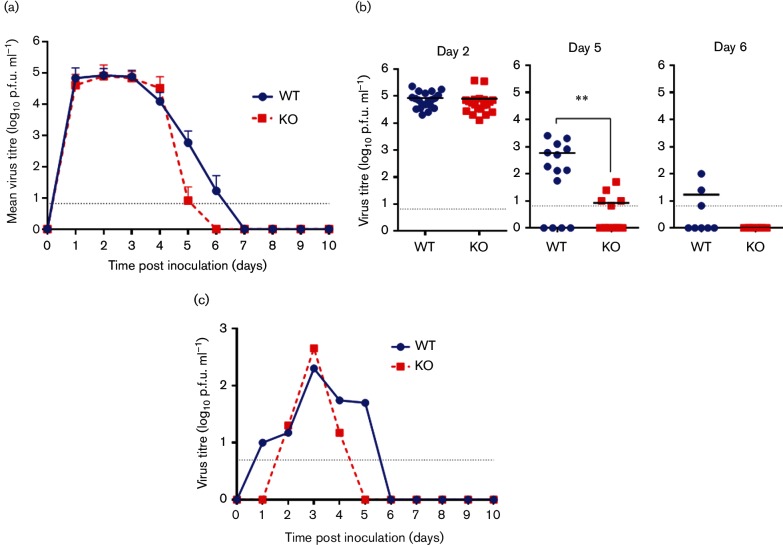
Virus shedding from the buccal cavity of high-dose-inoculated chickens and environmental viral titres. Infectious virus from groups of 20 chickens inoculated with 1×10^5^ p.f.u. (high dose) with either WT (blue circles) or KO (red squares) UDL01 influenza virus. Grey dotted baselines indicate limit of detection. (a) Mean infectious virus titres from buccal swabs. (b) Buccal shedding titres from individual birds at 2, 5 and 6 days post inoculation. (c) Infectious virus titres in communal drinking water. A pooled sample from the water sources in each isolator was assessed daily. Means±sd are shown where applicable. Levels of significance were based upon *P*-values from a Mann–Whitney U-test; **P<0.001.

To ascertain if the presence of a full-length PB1-F2 protein effected *in vivo* virus replication in chickens, we determined the level of infectious virus shed from both the buccal and cloacal cavities of birds inoculated with both high and low doses for 10 days following infection. All birds directly inoculated with either virus, WT or KO, robustly shed infectious virus from the buccal cavity commencing on day 1 post inoculation and there was no difference in the titre of infectious virus shed from the buccal cavity between the two groups up to day 4 post inoculation (Figs 4a and S1a). However, on day 5 post inoculation, for both viral doses, we observed that the majority of birds in the KO group had ceased shedding infectious virus from the buccal cavity and those that did shed virus had less than 100 p.f.u. ml^−1^ detectable [average p.f.u. ml^−1^ shed was 70 (±102) and 8 (±3) for the low and high dose, respectively]. In contrast at day 5 post inoculation, birds directly infected with the WT virus continued to shed significant levels of infectious virus from the buccal cavity [average p.f.u. ml^−1^ shed was 14 524 (±35 907) and 1082 (±1008) for the low and high dose, respectively] (Figs 4b and S1b). We detected sporadic shedding from the cloacal cavity of chickens inoculated with both the low and high viral doses of the WT and KO viruses and the number of birds shedding virus was lower in the KO (29 and 10 %, respectively) than the WT group (50 and 39 %, respectively) (Table 2).

**Table 2.** Cloacal shedding from chickens inoculated with WT and KO UDL01 influenza virus

Virus dose	Virus	% Birds shedding (*n*)	Mean shedding duration (days)*	Mean shedding titre (p.f.u.)†
High	WT	35 % (7/20)	1.57	1.67×10^3^ (±4.13×10^3^)
	KO	10 % (2/20)	1	1.50×10^3^ (±2.05×10^3^)
Low	WT	50 % (7/14)	1.14	2.76×10^3^ (±4.36×10^3^)
	KO	29 % (4/14)	1.25	1.74×10^2^ (±1.76×10^2^)

*Shedding assayed daily until 10 days post inoculation.

†Mean (±SD).

**Fig. 5.**
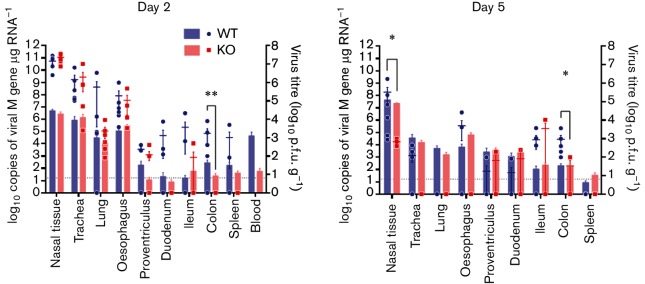
Viral titres in tissues harvested from high-dose-inoculated chickens. A panel of respiratory and gastrointestinal chicken tissues were harvested from the chickens inoculated with 1×10^5^ (high dose) of either WT (blue) or KO (red) UDL01 influenza virus. Tissues were harvested and processed at (a) 2 or (b) 5 days post inoculation. Copies of viral M gene vRNA (left axis) were determined via influenza specific qPCR on total tissue RNA (bars). Infectious virus (right axis) was determined by virus titration of homogenized chicken tissues (circles/squares). Grey dotted base lines indicate the limit of infectious virus detection. Means±sem were calculated from a minimum of three individual animals. Levels of significance were based upon *P*-values from Student *t*-tests; * *P*<0.05 for infectious virus, ∗∗*P*<0.05 for copies of viral mRNA.

The lower incidence of cloacal shedding of the KO virus from inoculated chickens led us to characterize the tissue tropism of the two viruses. Leymaire *et al.* (2014) have previously shown that the presence of a PB1-F2 protein increased the amount of vRNA detected in the intestine of birds inoculated with an HPAI virus at 2 days post inoculation. Using both qRT-PCR for vRNA and infectious virus particle titration, we determined the relative levels of the WT and KO viruses in a panel of respiratory and gastrointestinal tract tissues at both day 2 and 5 post inoculation for the high-dose infection study (Fig. 5). We found that in the respiratory tract (nasal turbinate, trachea and lungs) on day 2 there were no differences in either vRNA or infectious particle levels. The differences we observed in the respiratory tissues on day 5, higher levels of WT than KO virus in the nasal turbinate, were in line with the higher level of infectious particle shedding from the buccal cavity on day 5 by birds infected with the WT virus. In the gastrointestinal tract (oesophagus, duodenum, ileum and colon), we observed more virus in tissues from birds infected with the WT virus on both days 2 and 5 post inoculation, (Fig. 5), however, the increase was only statistically significant in the colon tissue samples.

### Increased environmental contamination by UDL01-WT virus

We measured the level of infectious virus in both the communal water and food sources shared by birds during the high-dose experiment. Whilst we found no infectious virus in the food source, even at the peak of viral shedding, we were able to isolate infectious virus from the common water source in the isolators, for both viruses (Fig. 4c). In the isolator that housed birds infected with WT virus, we detected virus from day 1 to 5 post inoculation; in contrast the KO virus was only detected on days 2–4 post inoculation, indicating that the presence of full-length PB1-F2 protein can lengthen the period of infectious environmental contamination for LPAI virus. PB1-F2 is believed to be a non-structural protein and several studies have failed to find it as a component of the influenza virus particle (Hutchinson *et al.*, 2015; Shaw *et al.*, 2008). Therefore, it is unlikely that an absence of PB1-F2 would affect virus particle stability. However, we did assess WT and KO infectious virus persistence in water at 25 °C (akin to the poultry isolator set up in which the *in vivo* experiments were conducted). No difference in stability of the infectious virus particles was seen (data not shown). There were also no HA sequence changes in samples of the viruses shed from both inoculated and in contact birds which also ruled out inherent stability differences between the viruses. The fact of increased environmental contamination has direct consequences for the efficiency of transmission to naive birds as the presence of PB1-F2 can lengthen the window of opportunity for naive birds to contract the virus from contaminated environmental sources, presumably through fomite/faecal-oral transmission routes.

### PB1-F2 increases the opportunity for in-contact transmission between chickens

**Fig. 6.**
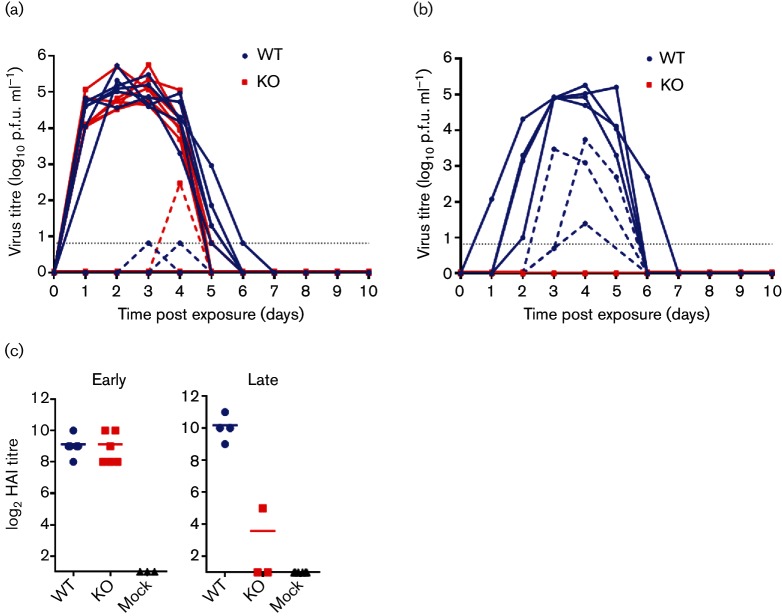
Transmission to naive contacts introduced at 1 and 5 days post inoculation. (a, b) Infectious virus titres in buccal (solid lines) and cloacal (dashed lines) swabs from naive contact chickens housed with either WT (blue) or KO (red) infected chickens. Contacts were exposed to infected chickens at either (a) early (1 day post inoculation) or (b) late (5 days post inoculation) time points. Grey baselines indicate the limit of detection. (c) HA inhibition titres on serum from contacts exposed to chickens inoculated with WT (blue circles) or KO (red squares) UDL01 at early (1 day post inoculation) or late (5 days post inoculation) time points. Serum was collected at 14 days post exposure and titred against the virus used for inoculation.

The difference in viral clearance dynamics and the environmental contamination period of the two viruses, that differed only by the presence of a PB1-F2 protein, lead us to investigate whether PB1-F2 had any effect on transmission parameters in co-housed chickens who shared a common water and food source. Transmission from directly inoculated chickens to in-contact birds at both early (24 h post inoculation) and later (5 days post inoculation) time points was assessed by the introduction of naive sentinels at these time points in separate experiments. Unsurprisingly, as no difference in buccal shedding between the WT and KO viruses was observed at early time points, naive sentinel animals that were introduced 24 h after the donor animals were directly inoculated, all became robustly infected independently of the virus to which they were exposed (Fig. 6a). Infection in the sentinel animals was determined via titration of infectious virus in buccal and/ or cloacal swabs and seroconversion 14 days post exposure (Fig. 6). In these early exposure contact animals, we saw a similar pattern to directly inoculated birds of prolonged viral shedding from birds infected by the virus containing a full-length PB1-F2 protein in contrast to earlier clearance of virus in those infected by the PB1-F2 KO virus (Fig. 6a). This experimental setup models a commercial poultry farming operation where large numbers of birds are continuously confined in close proximity.

To assess a more field-like environment experienced by the wild bird reservoir or poultry in a backyard flock, we introduced naive sentinel birds at 5 days post inoculation of the donor animals, when the disparity of buccal viral shedding in the donor birds was apparent. These birds were again co-housed and shared common water and food sources. The premise of this experimental setup was that in a free roaming environment birds can come into contact or share nourishment sources at any point during the viral infectious period since they are not strictly confined and their interactions are more transient. Therefore, initial contact at later time points during the infection period is important in the spreading of virus. We found that all birds in contact with those directly inoculated with a virus containing a full-length PB1-F2 (WT) became robustly infected, shedding virus from both buccal and cloacal cavities (Fig. 6b). In contrast, no viral shedding for any of the in-contact birds co-housed with those inoculated with the KO virus was detected (Fig. 6b). Serology performed on the in-contact birds confirmed there was also no seroconversion 14 days after exposure (Fig. 6c). This is the first time that the presence of a PB1-F2 protein has been demonstrated to affect transmission parameters in any species and suggests a scenario whereby it might be more favourable in an avian host for IAVs to encode for a full-length PB1-F2 protein.

## Discussion

The small PB1-F2 protein of IAVs is termed an accessory protein as it is not required for the basic replicative cycle of influenza in multiple host species (Pena *et al.*, 2012; Zamarin *et al.*, 2006). It has been established in several studies that there is a low aa evolutionary rate for the PB1 protein, necessary due to the vital structural and functional integrity of the viral polymerase protein; however, this results in a higher rate of aa evolution in the +1 reading frame and thus PB1-F2 (Trifonov *et al.*, 2009; Westgeest *et al.*, 2014). Despite this, there is an apparent unique pressure to maintain a full-length ORF of the protein in isolates that have been retrieved from avian species and this contrasts with the frequent truncation of the protein in mammalian host viral isolates by the incorporation of one or more stop codons (Krumbholz *et al.*, 2011; Zell *et al.*, 2007).

Analysis of 24 803 influenza PB1 segment sequences available on the NCBI database found that 93 % of viruses isolated from avian hosts maintained a full-length PB1-F2. Recently it has been reported that approximately 61 % of HPAI H5N1 isolates from 2013 had a truncated PB1-F2 (Kamal *et al.*, 2015); therefore we also examined if avian virus pathogenicity as defined by a multiple basic cleavage site (two or more basic aa), in the HA protein, was associated with PB1-F2 length. We found little variation in the frequency of full-length PB1-F2 in HPAI or LPAI viruses and a marginal reduction in HPAI (87.7 % HPAI vs 91.0 % LPAI) was entirely due to the H5 subtype (Table S3). An assessment of avian HA subtypes and full-length PB1-F2 conservation found that H5 (90 %), H6 (88 %), H9 (81 %), H13 (88 %) and H16 (83 %) subtypes all had lower percentages of full-length PB1-F2. A full analysis of all 16 avian HA subtypes is presented in Table S4.

Further analysis within the avian host grouping indicated that isolates from the order Galliformes (chickens, turkeys, quail, etc.) have a conserved full-length PB1-F2 at a slightly lower frequency of 87 % compared to the natural host reservoir bird orders of Anseriformes (ducks, geese, swans, etc.; 97 %) and Charadriiformes (shorebirds such as waders and gulls; 98 %) (Table 1) (Olsen *et al.*, 2006; Webster *et al.*, 1992). A full analysis of all avian orders can be found in Table S5. This suggested that PB1-F2 may contribute to a process more critical to the natural avian field environment than an intensive poultry rearing scenario. An obvious difference between these two avian settings is the modes and dynamics of transmission of IAVs.

The aim of this study was to interrogate fully the contribution that PB1-F2 makes to viral fitness in an avian host. We assessed viral infectivity, pathogenicity and transmissibility between chickens of isogenic viruses differing only by the presence of a full-length PB1-F2 protein.

Two other published studies have undertaken a similar approach to understanding the role of PB1-F2 in the context of avian species infection with influenza viruses (Leymarie *et al.*, 2014; Schmolke *et al.*, 2011). However, both focused solely on the effect that a full-length PB1-F2 protein has on the pathogenic nature on H5N1 viruses which were already highly pathogenic. The study by Schmolke *et al.* (2011) assessed the effect of knocking out the PB1-F2 protein on the pathogenicity of H5N1 in White Pekin ducks. No effect on pathogenicity was observed, but an increased systemic spread of the virus in the ducks was demonstrated for HPAI H5N1 virus containing a full-length PB1-F2 in contrast to when PB1-F2 expression was absent (Schmolke *et al.*, 2011). Leymaire *et al.* (2014) considered the effect of a full-length PB1-F2 on the pathogenicity of an HPAI H5N1 virus in the chicken model and in accordance with our findings presented here, found that removal of full-length PB1-F2 actually enhanced pathogenicity (when examined at doses at which WT virus is not 100 % lethal). This study also found increased viral tissue dissemination both *in ovo* of 10-day-old chicken embryonated eggs and in experimentally inoculated chickens when PB1-F2 protein was present (Leymarie *et al.*, 2014). Recently, another study that investigated the effect of PB1-F2 on transmission in turkeys concluded that lack of a full-length PB1-F2 gene in a triple-reassortant H3N2 swine influenza virus increased histopathology in turkeys and provided a transmission advantage (Deventhiran *et al.*, 2015). While the increase in pathogenicity when PB1-F2 is absent is in line with Leymaire *et al.* (2014) and our study the transmission advantage of a virus lacking PB1-F2 differed to our finding. Transmission in the Deventhiran *et al.* (2015) study was not robustly assessed and was determined on a single clinical sign (diarrhoea) and weak positive seroconversion; no virus was detected from any of the in-contact birds. In addition, differences in the genetic history of the virus isolates studied, and/or inherent pathogenicity may account for these differences.

In our study we demonstrate that, in an LPAI background, the presence of a PB1-F2 protein suppressed pathogenicity in a chicken model. The increased pathogenicity observed between days 4 and 5 post inoculation of the birds was preceded by the observation of increased viral dissemination *in vivo* on day 2 post inoculation. Virus was recovered more frequently, and in higher titres, from the gastrointestinal tract of chickens when a full-length PB1-F2 protein was present. The intrinsic pathogenicity of a virus could also impact on its transmission potential (Cho *et al.*, 2007). A pathogenic virus which can induce a great clinical impact on an infected host has the potential to modulate the infected subject’s and their contacts’ behaviour in a manner that would reduce interactions: hiding away and lethargy. In contrast during a low or non-pathogenic viral infection, the mixing of hosts may be more likely.

In Pakistan and Southeast Asian countries where H9N2 LPAI virus is enzootic, informal poultry farming accounts for approximately 30 % of total poultry production (Conan *et al.*, 2012; Tufail *et al.*, 2012). In this setting it is rare for birds to be densely packed together for prolonged periods of time with birds transiently interacting or sharing resources, limiting the time and incidence of direct transmission between hosts. In these natural field conditions, poultry transmission of influenza viruses is thought to occur predominately through the faecal-oral route by ingestion of virus from contaminated food and water sources. It is therefore important that influenza virus is maintained in the environment for effective transmission to occur (Brown *et al.*, 2007; Hinshaw *et al.*, 1979). Transmission is an area of influenza biology which has not previously been robustly investigated in relation to PB1-F2.

Importantly, in this study we show that the PB1-F2 protein prolongs viral shedding from infected chickens and that this has the effect of increasing the window of opportunity for transmission to naive birds. Increased cloacal shedding of the UDL01-WT virus in comparison to the UDL01-KO virus was detected which complemented the observation that more WT virus was present in gastrointestinal tissues of infected birds. In addition, buccal shedding of the virus was sustained for 1–2 more days from chickens infected with the virus containing full-length PB1-F2. The increase of both buccal and cloacal viral shedding in the WT-infected birds resulted in longer environmental contamination of the chicken’s water source during these studies. The alteration of these parameters by the presence of a full-length PB1-F2 resulted in transmission at a later time point (5 days post inoculation) for the UDL01-WT, whereas no transmission at this time point was observed for the UDL01-KO virus.

Our results point to a role for PB1-F2 in enhancing the transmission potential of IAVs in the natural avian reservoir or in backyard flock environments rather than in close living commercial poultry farms. This aligns with the avian sequence data which showed that PB1-F2 length is conserved at a high frequency in the influenza virus reservoir species Anseriformes and Charadriiformes than poultry (Galliformes) despite genetic pressure on the +1 reading frame of the essentially conserved viral polymerase segment. Poultry samples in the NCBI database are likely isolated from intensively farmed poultry where the role of PB1-F2 in transmission is less important than the free living natural reservoir aquatic birds.

In summary, we conclude that the IAV accessory protein PB1-F2 enhances pathogenicity and transmission of avian influenza viruses in avian hosts. This work emphasizes the need to understand host-specific differences in terms of both genetics and behaviour when analysing the role of viral proteins.

## Methods

### Ethics statement.

All animal work was approved and regulated by the UK government Home Office under the project licence (PPL 30/2952). All personnel involved in the procedures were licensed by the UK Home Office. Euthanasia of chickens was carried out by intravenous administration of sodium pentobarbital and confirmed through cervical dislocation.

### Viruses and cells.

Recombinant A/chicken/Pakistan/UDL01/08 H9N2 virus (UDL01-WT) was generated using reverse genetics as previously described (Hoffmann *et al.*, 2000). UDL01-KO whereby a stop codon was introduced at aa position 12 in the PB1-F2 ORF was constructed by site-directed mutagenesis in the PB1 genetic segment prior to rescue. Virus stocks were produced via passage in 10-day-old embryonated chicken eggs; the allantoic fluid was harvested after 48 h and titrated by plaque assay on Madin–Darby canine kidney (MDCK) cells (ATCC).

MDCK and human embryonic kidney (HEK) 293T cells (ATCC) were maintained in Dulbecco's modified Eagle's medium (DMEM) (Gibco-Invitrogen) supplemented with 10 % FBS (Biosera), 1 % penicillin/streptomycin (Sigma-Aldrich) and 1 % non-essential aa (Sigma-Aldrich). Primary chicken kidney cells (cKCs) were prepared in house as previously described (Penzes *et al.*, 1994) from 2–3-week-old Rhode Island Red chickens and maintained in EMEM, 7 % BSA (Sigma), 1 % penicillin/streptomycin and tryptose phosphate broth. Both MDCKs and cKCs were maintained at 37 °C with 5 % CO_2_. *Ex vivo* chicken tracheal organ cultures (cTOCs) were prepared as previously described (Armesto *et al.*, 2009) and maintained in EMEM supplemented with 1M HEPES and 1 % penicillin/streptomycin; these were maintained under liquid at 37 °C with no addition of CO_2_.

### Validation of segment 2 gene expression.

To analyse viral gene expression from transfected plasmids, a previously published strategy was adopted (Wise *et al.*, 2011). Briefly, nucleotides 1–380 of UDL-1/08 PB1 were cloned into AgeI/KpnI sites of pEGFPN1 (Clontech). Using site-directed mutagenesis the position of the EGFP ORF was adjusted into frame with either the PB1 or PB1-F2 reading frames, while concurrently removing the GFP ATG codon. Additional segment 2 mutations as detailed in the results section were made using site directed mutagenesis with the WT segment plasmids as templates. 2 µg of each plasmid was transfected into HEK 293T cells using lipofectamine 2000 (Invitrogen) according to the manufacturer's instructions. 12 h after transfection, 10 µM MG132 (Sigma-Aldrich) was added to the medium for a further 12 h. Cells were harvested and the presence of alpha-tubulin and PB1-, PB1-N40- and PB1-F2-GFP was detected via Western blot using rabbit anti-alpha tubulin (Abcam) and mouse anti-GFP (Gene Tex) antibodies with IRDye 800CW/IRDye 680RD secondary antibodies (LI-COR) and visualized using the Odyssey CLx (Li-Cor). Densitometric analysis was performed using Image Studio software (Li-Cor) from three independent experiments and the intensities for PB1-, PB1-N40- and PB1-F2-GFP were normalized to alpha-tubulin. Fold change in protein expression relative to the WT was calculated.

### Multi-cycle growth curves.

MDCK cells and cKCs were infected with virus m.o.i. of 0.0001 at 37 °C for 1 h, in triplicate. The cells were washed and incubated at 37 °C in serum-free DMEM containing 2 µg ml^−1^ TPCK trypsin (Sigma-Aldrich). A 500 µl aliquot of supernatant was taken 24, 48 and 72h after infection and stored at −80 °C and 500 µl of fresh medium was added back to each well. Virus was titrated by plaque assay.

Five cTOCs per time point were inoculated with 100 p.f.u. of virus at 37 °C for 1 h in cTOC maintenance medium. cTOCs were washed then placed in glass tubes for rotation at 37 °C in 1 ml of maintenance medium. Medium was harvested from five individual cTOCs at 24, 48 and 72 h for each virus. The virus in the supernatants was titrated by plaque assay.

To perform growth curves *in ovo*, three 10-day-old embryonated hens’ eggs per virus, per time point, were inoculated into the allantoic cavity with 100 p.f.u. of virus. Eggs were incubated at 37 °C for 24, 48 or 72 h; the allantoic fluid was harvested and titrated by plaque assay.

### Titration of infectious virus.

Infectious virus in growth curves, chicken swabs, environmental samples or homogenized tissue was titrated by plaque assay on MDCK cells. MDCK cells were inoculated with 10-fold serially diluted samples and overlaid with 0.6 % agarose (Oxoid) in supplemented DMEM (1× MEM, 0.21 % BSA V, 1 mM l-glutamate, 0.15 % sodium bicarbonate, 10 mM HEPES, 1× penicillin/streptomycin (all Gibco) and 0.01 % Dextran DEAE (Sigma-Aldrich), with 2 µg ml^−1^ TPCK trypsin (Sigma-Aldrich). They were then incubated at 37 °C for 72 h. Plaques were developed using crystal violet stain containing methanol. The limit of virus detection in the plaque assays was 6.67 p.f.u. ml^−1^.

### Minimum lethal dose and minimum infectious dose assays.

Groups of five, 10-day-old embryonated hens’ eggs were inoculated into the allantoic cavity with 100 µl of 10-fold decreasing concentrations of each virus in PBS from 10 000 to 0.001 p.f.u. The embryos were monitored in a double-blinded study by two people, twice daily until 72 hpi or until signs of end point as defined by lack of embryo movement and/or severe haemorrhage. Eggs were chilled at 4 °C to confirm death and the allantoic fluid was harvested and presence of infectious virus determined. The influenza viral infectivity assay was performed by inoculating a 96-well plate of MDCK cells with 100 µl of undiluted allantoic fluid for 1 h at 37 °C. Following washing in PBS, serum-free DMEM was added to the cells for 12 h. Cells were fixed with 50 % methanol : 50 % acetone and immune-stained for influenza nucleoprotein (NP). NP was detected using a mouse α-NP antibody produced at The Pirbright Institute and an IRDye 800CW α-mouse secondary antibody (Li-Cor) and visualized using Odyssey Imaging Systems (Li-Cor). The MLD was defined as the lowest viral dose resulting in death of all infected embryonated chicken eggs and the MID defined as the lowest viral dose required to produce infection in all embryonated chicken eggs.

### Infection and transmission of virus in chickens.

Mixed, sex-specific pathogen free (SPF) Rhode Island Red chickens bred at The Pirbright Institute poultry production unit were used at 3 weeks old. Pre-infection sera were obtained from all chickens and all were negative for reactive influenza antibodies against A/chicken/Pakistan/UDL01/08 (UDL01). Chickens were split into three groups: Mock, UDL01-WT and UDL01-KO. The UDL01-WT and UDL01-KO were housed in self-contained BioFlex B50 Rigid Body Poultry isolators (Bell Isolation Systems). Chickens were inoculated intranasally with 100 µl (50 µl nare^−1^) of PBS for Mock group or UDL01-WT or UDL01-KO either at a low dose of virus (5×10^3^ p.f.u.) or high dose (1×10^5^ p.f.u.). Naive sentinel birds were introduced into the isolators with infected birds 24 h or 5 days after direct inoculation to assess transmission. Birds were humanely euthanized 14 days post inoculation and sera samples collected for analysis of virus specific antibodies.

### Sample collection from chickens and the isolator environment.

Infection of birds was assessed by swabbing both the buccal and cloacal cavities daily with sterile polyester tipped swabs (Fisher Scientific) which were transferred into viral transport medium (WHO, 2006), vortexed briefly, clarified and stored at −80 °C prior to virus detection.

On days 2 and 5 post inoculation, four birds from each group were humanely culled and organs (nasal tissue, trachea, lungs, oesophagus, proventriculus, duodenum, ileum, colon, blood and spleen) were snap frozen. Prior to processing, the organs were thawed on ice and homogenized inside a tube containing a 5 mm steel bead in a Bead Mill (Retsch MM 300) under the conditions of 20 Hz for 4 min. For infectious virus quantification, 20 % w/v of organ to PBS containing 1 % penicillin/streptomycin was added to the tube prior to homogenization; this was then clarified by centrifugation and the supernatant was used in plaques assays.

For environmental sample collection, approximately 1 ml of drinking water was collected from both water sources inside each isolator. To the sample, 10× DMEM was added to achieve 1× DMEM. Approximately 1 g of dry food was added to 1 ml of viral transport medium and vortexed briefly. Both environmental sample types were clarified of debris by centrifugation at 450 ***g*** for 2 min and stored at −80 °C prior to virus detection by plaque assay.

### qRT-PCR of viral M gene.

For viral M gene detection from chicken tissue, 750 µl of Trizol LS (Life Technologies) was added prior to homogenization. RNA was extracted from homogenized tissues by chloroform extraction and purification with a RNeasy kit (Qiagen). Quantification of vRNA copies was undertaken using a single-step real-time reverse transcription PCR, the Superscript III Platinum One-Step qRT-PCR Kit (Life Technologies). Primers and a TaqMan probe specific for a conserved region of the influenza A matrix gene were used as described previously (Spackman *et al.*, 2002). Cycling conditions were: 50 °C, 5 min; 95 °C, 2 min and then 40 cycles of 95 °C, 3 s and 60 °C, 30 s, using a 7500 fast real-time PCR machine (Applied Biosystems). A T7 transcribed RNA standard of the M gene was run alongside each assay to generate a standard curve.

### Genome: p.f.u. ratios.

Three independently produced viral stocks in embryonated chicken eggs for each virus were assessed. The p.f.u. were determined using standard plaque assay on MDCK cells. In parallel, RNA was extracted and the number of influenza M gene copies determined via qRT-PCR as previously described. The genome : p.f.u. ratio was calculated and the mean for each virus reported with sd.

### Statistical analysis.

All statistics were performed using the statistical function on GraphPad Prism 7 (GraphPad Software). Statistical calculations included two-tailed paired Student’s *t*-test and Mann–Whitney U-test. *P*-values <0.05 were considered significant.

### Bioinformatics.

In June 2015, all sequences were downloaded from the NCBI Influenza Virus Database (http://www.ncbi.nlm.nih.gov/genomes/FLU/Database/nph-select.cgi?go=database) as full-length DNA sequences; we excluded identical sequences through the collapse function. All sequences were analysed using mega6: Molecular Evolutionary Genetics Analysis version 6.0 (Tamura *et al.*, 2013). For PB1-F2 length analysis, full-length PB1 sequences were downloaded and the PB1-F2 ORFs were extracted, aligned and translated. Only sequences with methionine at the canonical aa position zero were analysed to only include C-terminal truncations. For species analysis, separate sequence sets were downloaded for human, swine and avian. The avian orders and specific subtypes were categorized from total avian sequences after download. For analysis of PB1-F2 by pathogenicity, matched full-length HA and PB1 sequences were downloaded. The sequence surrounding the cleavage site of HA was extracted, aligned and translated. Viruses were classified as HPAI if they contained >2 basic aa (Arg, Lys or His) upstream, proximal to the cleavage motif as defined previously (OFFLU, 2014).

## Supplementary Data

301 Supplementary File 1
