# Supplementary material for: Influenza A virus PB1-F2 protein prolongs viral shedding in chickens lengthening the transmission window
Source: J Gen Virol. 2016 Oct 13;97(10):2516–27. doi: 10.1099/jgv.0.000584 (PMC5078828; doi:10.1099/jgv.0.000584)
Supplement: Supplementary File 1 [file jgv-97-2516-s001.pdf]

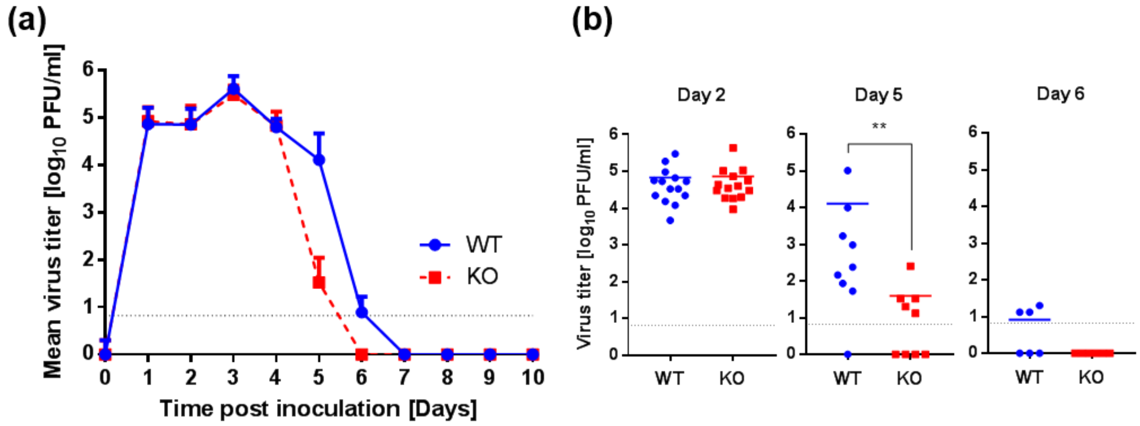

Fig S1. Virus shedding from the buccal cavity of low dose inoculated chickens and environmental viral titres.

Infectious virus from groups of 20 chickens inoculated with 5 x 10^3 pfu (low dose) with either WT (Blue circles) or KO (red squares) UDL01 influenza virus. Grey dotted baselines indicate limit of detection. (a) Mean infectious virus titres from buccal swabs. (b) Buccal shedding titres from individual birds at 2, 5 and 6 days post inoculation. Means ±SD are shown. Levels of significance were based upon P-values from a Mann-Whitney U-test; **P<0.001.

Table S1. Percentage of influenza A sequences possessing PB1-F2 ≥79aa, ≥87aa or ≥90aa isolated from avian, human and swine hosts.

Host	Number of sequences analysed	% sequences with PB1-F2 ≥ 79aa	% sequences with PB1-F2 ≥ 87aa	% sequences with PB1-F2 ≥ 90aa
**Avian***	10401	96	93	93
Anseriformes	6917	97	97	94
Galliformes	1804	92	87	85
Charadriiformes	1298	98	98	97
**Human**	11729	43	42	42
**Swine**	2673	56	48	48
**Total**	24803	67	65	64

*All avian orders

Table S2. Minimum infectious dose and minimum lethal dose of UDL-1 WT and KO in chicken embryos

Virus	Minimum Infectious Dose (Pfu)[a]	Minimum Lethal Dose (Pfu)[b]	Viral M gene RNA copy number / ml allantoic fluid*#	Viral genome: pfu ratio
WT	0.1	1	5.08×10^{7} ($\pm 2.82 \times 10^{7}$)	14.5 (±2.13)
KO	0.1	0.01	7.29×10^{7} ($\pm 3.11 \times 10^{7}$)	12.39 (±4.37)

[a] The lowest dose required to cause infection in 100% of eggs receiving that dosage by 72 hours post infection.

[b] The lowest dose required to cause 100% mortality in all infected eggs receiving that dosage by 72 hours post infection.

*100 pfu inoculation.

[#] Mean (± standard deviation).

Table S3. Percentage of influenza A possessing full-length (≥87aa) PB1-F2 isolated from LPAI and HPAI.

Virus Subtype	Pathogenicity*	% ≥87aa PB1-F2 (n)
**H5 and H7**	HPAI[a]	88 (1213)
	LPAI[b]	91 (1508)
	All Sequences	90 (2721)
**H5**	HPAI	87 (1104)
	LPAI	94 (487)
	All Sequences	89 (1591)
**H7**	HPAI	100 (109)
	LPAI	90 (1021)
	All Sequences	91 (1130)

*Pathogenicity determined by presence or absence of a polybasic site (>two basic amino acids) in the Hemagglutinin cleavage site

[a] Highly Pathogenic avian influenza

[b] Low Pathogenicity avian influenza

Table S4. Distribution of different length PB1-F2 genes encoded by avian isolated influenza viruses arranged by hemagglutinin subtype.

PB1-F2 Length (aa)	% length PB1-F2 by Hemagglutinin Subtype															
	**1***	**2**	**3**	**4**	**5**	**6**	**7**	**8**	**9**	**10**	**11**	**12**	**13**	**14**	**15**	**16**
**Sequences (n)**	529	404	1323	1278	2222	1194	1404	139	1000	787	471	158	80	14	9	30
**8**		0.3		0.1	0.5	0.3	0.6			0.5			1.3			
**11**	2.5	0.3	0.1	0.1	1.9	1.3	0.1		1.4							
**24**					0.3	0.1			0.1							
**25**	0.2	0.5		0.1	0.3	0.2	1.1		2.7	0.1	0.2					
**27**				0.2					0.1							
**34**					0.1	4.1	3.3		2.9	4.8	0.4					
**56**					0.6	3.4			0.1							
**57**		0.3	0.1	0.1	5.8	1.6	2.4		1.3				11.3			16.7
**63**					0.1	0.6										
**76**					0.1	0.2	0.9		0.3							
**79**			0.5		0.3	0.3	0.1		10.2							
**87**	3	2.7	2.6	2.9	2	4.8	1.1	4.3	1.4	2.3	4.5	1.3				
**90**	94.3	96.0	96.7	96.2	87.8	83.1	90.2	95.7	78.7	91.2	94.9	98.7	87.5	100	100	83.3
**101**			0.2	0.3	0.4	0.2	0.3		0.8	1						
**≥79**	97.4	98.8	99.9	99.5	90.5	88.3	91.7	100	91.1	94.5	99.4	100	87.5	100	100	83.3
**≥87**	97.4	98.8	99.4	99.5	90.1	88	91.6	100	80.9	94.5	99.4	100	87.5	100	100	83.3
**≥ 90**	94.3	96	96.8	96.6	88.2	83.3	90.5	95.7	79.5	92.3	94.9	98.7	87.5	100	100	83.3

* Hemagglutinin subtype

Table S5. Percentage of influenza A sequences possessing a full length PB1-F2 ≥87aa isolated from individual avian orders.

Host	Number of sequences analysed	% sequences with PB1-F2 ≥ 87aa
All Orders	10401	93
Accipitriformes	24	96
Anseriformes	6928	97
Charadriiformes	1298	98
Ciconiiformes	37	100
Columbiformes	31	87
Galliforms	1804	85
Gruiformes	16	94
Pelecaniformes	13	85
Podicipediformes	17	88
Psittaciformes	5	80
Ratites	21	100
Suliformes	2	100
